# Contrast enhancement of stimulus intermittency in a primary olfactory network and its behavioral significance

**DOI:** 10.1186/jbiol120

**Published:** 2009-02-20

**Authors:** Hong Lei, Jeffrey A Riffell, Stephanie L Gage, John G Hildebrand

**Affiliations:** 1 ARL-Division of Neurobiology, University of Arizona, Tucson, AZ 85721-0077, USA

## Abstract

**Background:**

An animal navigating to an unseen odor source must accurately resolve the spatiotemporal distribution of that stimulus in order to express appropriate upwind flight behavior. Intermittency of natural odor plumes, caused by air turbulence, is critically important for many insects, including the hawkmoth, *Manduca sexta*, for odor-modulated search behavior to an odor source. When a moth's antennae receive intermittent odor stimulation, the projection neurons (PNs) in the primary olfactory centers (the antennal lobes), which are analogous to the olfactory bulbs of vertebrates, generate discrete bursts of action potentials separated by periods of inhibition, suggesting that the PNs may use the binary burst/non-burst neural patterns to resolve and enhance the intermittency of the stimulus encountered in the odor plume.

**Results:**

We tested this hypothesis first by establishing that bicuculline methiodide reliably and reversibly disrupted the ability of PNs to produce bursting response patterns. Behavioral studies, in turn, demonstrated that after injecting this drug into the antennal lobe at the effective concentration used in the physiological experiments animals could no longer efficiently locate the odor source, even though they had detected the odor signal.

**Conclusions:**

Our results establish a direct link between the bursting response pattern of PNs and the odor-tracking behavior of the moth, demonstrating the behavioral significance of resolving the dynamics of a natural odor stimulus in antennal lobe circuits.

## Background

An animal's nervous system must encode environmental stimuli that are important for the individual's survival and reproduction. According to a generally accepted coding theory, neural-discharge patterns, not the action potential itself, carry information about specific stimulus features [1]. Searching for behaviorally relevant patterns of neuronal activity has proved to be challenging, however, owing to the difficulty of identifying those activities that are directly responsible for natural behaviors or perceptions [2].

Although specific coding questions differ for different sensory systems, the conceptual issues are similar. For the olfactory system, an important task is to resolve the spatiotemporal dynamics of olfactory stimuli. In nature, odor molecules released from a source form an odor plume with a dynamic, intermittent structure due to turbulent movement of the fluid [3]. Animals navigating in such odor plumes therefore are exposed to intermittent olfactory stimulation, which is further aided by the animal's movement in the plume [4,5]. The behavioral importance of stimulus intermittency has been demonstrated clearly through work with insects, in particular moths, where discontinuous stimulation is required for successful odor-source-seeking behavior [6-10]. Results from further studies in moths and other insects detail a nearly universal strategy for odor-source location, that is, upwind locomotion modulated by moment-to-moment encounter with individual odor filaments, with each encounter resulting in an upwind surge [11-14]. These findings suggest that stimulus intermittency is a critical feature that must be resolved with high fidelity by the insect's olfactory system.

Extensive previous work on the sex-pheromonal communication system of moths makes it a useful model for studying olfactory processing of stimulus intermittency [15]. When a flying male moth or a walking insect [16] encounters a pheromone-laden filament, chemosensory information about that stimulus is relayed by olfactory receptor cells (ORCs) in the male's antennae [17] to a specialized region of the antennal lobe (AL; the analog of the olfactory bulb in vertebrates) – the male-specific macroglomerular complex (MGC), situated near the entrance of primary-sensory axons into the AL [18]. The projection (output) neurons (PNs) of the MGC (MGC-PNs), which relay information about sex-pheromonal stimulation to higher centers in the brain, have been shown to respond to pulses of pheromone delivered at a rate of up to 10 per second, with bursts of action potentials interspersed with periods of inhibition [19-21]. An implicit assumption is that the behavioral efficacy of stimulus intermittency depends on such bursting neural responses of PNs. This hypothesis, however, has never been tested directly. Here we used a juxtacellular recording technique [22] in conjunction with pharmacological manipulation and found that a GABA_A_-receptor antagonist, bicuculline methiodide (hereafter called bicuculline), reliably and reversibly disrupted the ability of MGC-PNs to encode intermittent pheromone pulses. While having no significant effect on the sensitivity of MGC-PNs in detecting pheromone, bicuculline injected into the MGC of both ALs caused the moth to navigate ineffectively in a turbulent (or intermittent) odor plume.

## Results

### Effects of bicuculline on the firing pattern of MGC-PNs

**Figure 1:**
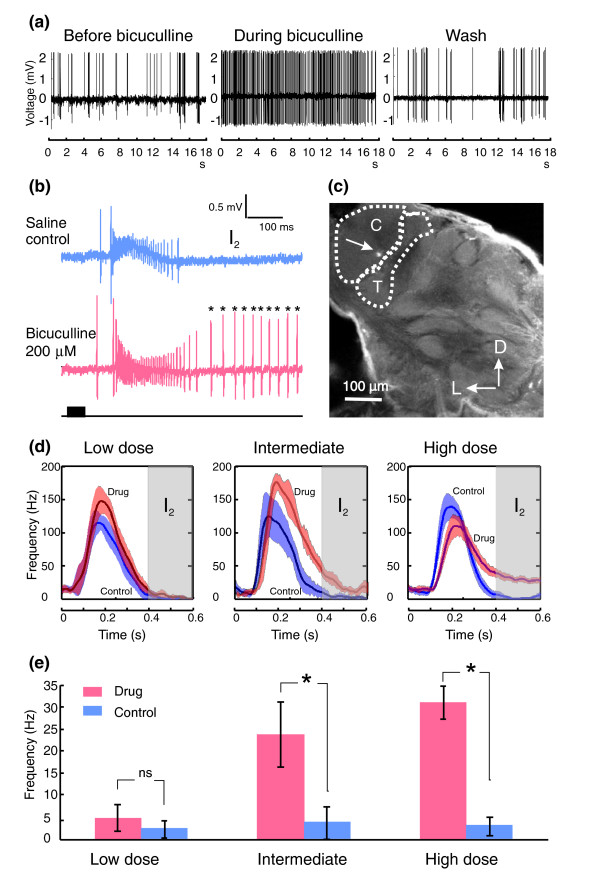
Effects of bicuculline on the firing pattern of MGC-PNs. **(a) **Shown as raw spike traces, bath application of 100 μM bicuculline changed the spontaneous firing pattern of an MGC-PN from a random bursting (left) to a more regular tonic pattern (middle). This change was reversed with saline wash (right). **(b) **The M inhibitory period (I_2_) that typically follows the odor-evoked excitatory phase in MGC-PNs (upper panel) was completely blocked by treatment with 200 μbicuculline, resulting in an extended excitatory response (asterisks, lower panel). Odor pulse is indicated by the black bar below the traces. **(c) **Confocal micrograph showing the lucifer yellow fluorescent mark (arrowed) in the cumulus (C) deposited by the glass electrode used to record the C-PN in (b). T, toroid I. **(d) **Graphs of peristimulus responses (derived from five odor pulses) of 25 MGC-PNs to their specific ligands under saline control (blue curve; mean ± SEM) and bicuculline treatment (orange curve; mean ± SEM) at low (25 μM, *n* = 8), intermediate (50 μM or 100 μM, *n* = 7), and high (200–500 μM, *n* = 10) dosages. The onset of the 50 ms stimulus was at time zero. **(e) **Histograms derived from the graphs in (d). The shaded areas represent the I_2 _period, during which the averaged firing rate was not significantly different (NS) between low-dose bicuculline treatment and saline control, but was significantly elevated by intermediate and high-dosage bicuculline treatment. The abbreviation ns and the asterisks respectively indicate non-statistical (Mann Whitney U test, *p *> 0.05 for low dose, *n* = 8) and statistical significance (Mann Whitney U test, *p *< 0.03 for intermediate dose, *n* = 7; *p *< 0.001 for high dose, *n* = 10).

This study focused on MGC-PNs with dendritic arborizations confined to one of the two main glomeruli of the MGC, the cumulus (C-PNs) or toroid I (T-PNs) [23]. These PNs are readily identifiable through their response specificity and pattern, and were further verified by the electrode location (Materials and methods). MGC-PNs were spontaneously active, randomly generating brief bursts of spikes (minimum of 3 spikes). In the example shown in Figure 1a, the average frequency of bursts was around 0.6 per second. The duration of the inter-burst intervals was variable, ranging from a few hundred milliseconds to a few seconds (mean ± SEM: 1.08 ± 0.13 s). In all PNs (*n* = 25), bath application of bicuculline apparently changed the spontaneous activity pattern from randomly bursting to tonic firing, during which the inter-spike interval (ISI) was about 140 ms (139.5 ± 19.7 ms; mean ± SEM, *n* = 25) and the coefficient of variation (CV) of the ISI was significantly lower (1.33 ± 0.089; mean ± SEM, *n* = 25) than that during the pre-drug period (*t *test: *p *< 0.001; 1.58 ± 0.074; mean ± SEM, *n* = 25) (Figure 1a; supplemental Figure 1a–c in Additional data file 1). It took about 20 minutes to observe significant changes caused by drug application (supplemental Figure 1a,b in Additional data file 1). Interestingly, the tonic firing periods were intermixed with non-spiking periods of similar length (supplemental Figure 1c,d in Additional data file 1). The drug effect could be completely reversed after washing-out with physiological saline for about 30 minutes (Figure 1a; supplemental Figure 1a,b in Additional data file 1). These obvious changes in spontaneous firing patterns allowed us to determine unambiguously when bicuculline had exerted its full effect on the PNs, thus allowing us to time the stimulus delivery before, during and after drug application.

The neuron in Figure 1b had the stereotypical response profile of C-PNs, with excitatory response to C15, a chemical mimic of a key component of the sex pheromone of *M. sexta*, *E*10,*E*12,*Z*14-hexadecatrienal [24], and inhibitory response to Bal (or bombykal, *E*10,*Z*12-hexadecadienal), the second key component [25]. The excitatory phase was immediately followed by a typical after-hyperpolarization phase I_2 _(Figure 1b, upper panel; supplemental Figure 2a in Additional data file 1). Moreover, a dye-marking technique (Materials and methods) revealed the location of the recording electrode in the cumulus (Figure 1c). During the bicuculline application (200 μM) the spiking activity was extended into the normally silent I_2 _period (Figure 1b, asterisks in the lower panel; supplemental Figure 2b in Additional data file 1), suggesting that the mechanisms underlying I_2 _were disrupted by bicuculline. Most of the 25 bicuculline-treated MGC-PNs at moderate (50 or 100 μM) or high (200 or 500 μM) concentrations showed such extended spiking responses, resulting in a significantly elevated firing rate during the I_2 _period (Figure 1d,e; Mann Whitney U test, *p *< 0.03 for intermediate dose, *n* = 7; *p *< 0.001 for high dose, *n* = 10). At a lower concentration (25 μM), the I_2 _period did not differ significantly from the control (Mann Whitney U test, *p *> 0.05, *n* = 8). Interestingly, the peak firing rate during the response decreased with increased drug dosage; however, it was not statistically significant when compared with the saline control (Figure 1d).

**Figure 2:**
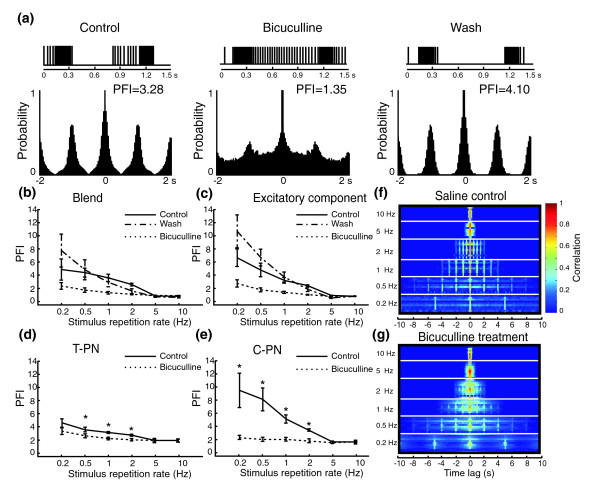
Bicuculline-effects on PNs' pulse-tracking capability. **(a) **Autocorrelation-based pulse-following index (PFI) was calculated to quantify the capability of PNs to track odor pulses delivered at 1 Hz repetition rate under saline control (left), bicuculline treatment (center), and saline wash (right). The raster plots above the correlograms illustrate the response of a T-PN to two consecutive odor pulses. Note that the drop in PFI value during bicuculline treatment is consistent with the decreased pulse resolution shown in the raster plots. **(b-e) **Population data (mean ± SEM) showing that bicuculline treatment consistently decreases the PFI values. (b, c) This effect was independent of stimulus type: (b) blend; (c) individual excitatory stimulus component. However, the PFI profiles for (d) T-PNs and (e) C-PNs were dramatically different, with C-PNs having higher PFI values in the range 0.2–1 Hz than the T-PNs under saline control (solid line), thus resulting in a greater drop in PFI values from control to bicuculline treatment (dotted line). Asterisks indicate statistical significance between control and drug treatment (repeated-measure two-way ANOVA at *p *= 0.05 level). **(f-g) **Stacked correlograms derived from the responses of ten PNs to their specific ligands show their capability to track odor pulses delivered at various repetition rates (ranging from 0.2 to 10 Hz) under (f) saline control and (g) bicuculline treatment. The pseudocolor scale, indicating the correlation coefficient, applies to both panels.

One potential consequence of the bicuculline-caused prolonged excitation was to decrease the contrast between the excitatory phase and the I_2 _period, thus resulting in a compromised coding of intermittent odor pulses. Comparing a PN's reliability in tracking odor pulses with or without bicuculline supported this idea (supplemental Figure 2b in Additional data file 1). Another example is shown in Figure 2a. Under saline control this neuron generated bursts of spikes locking onto each of the five odor pulses delivered at a rate of one pulse per second. Two consecutive bursting responses were illustrated with raster plots (Figure 2a, left, upper panel). The silent I_2 _period clearly followed the excitatory phase until the spontaneous activity resumed. To quantify the PN's ability to follow the repeated odor pulses, the odor-driven bursting responses were assessed with auto-correlation analysis, which revealed periodic peaks separated by 1-s intervals (Figure 2a, left, lower panel). These intervals directly correspond to the inter-pulse interval of the odor stimuli. Furthermore, an autocorrelogram-based pulse-following index (PFI) was calculated to reflect the ratio between the peak correlation at a specified time lag (for example, 1 s for 1 s^-1 ^pulse train, 2 s for 0.5 s^-1 ^pulse train) and the averaged correlation between the central peak and the specified peak (Materials and methods). The higher the PFI, the better the PN resolved pulses. During bicuculline application, the silent I_2 _period was filled with spikes, which resulted in a much-deteriorated periodicity in the autocorrelogram (Figure 2a, center). Consequently the PFI was reduced 59% from 3.28 for the saline control to 1.35 for the drug treatment. The bicuculline-induced changes could be reversed by washing the preparation with saline solution (Figure 2a, right), resulting in a slightly higher PFI than the control (4.10 versus 3.28), probably as a result of reduced background firing. The averaged PFIs among the ten bicuculline-treated PNs were significantly lower than that during the saline control on almost every stimulus repetition rate (Figure 2b,c, dotted lines). Two-way repeated-measures ANOVA [26] on the control and drug-treatment data showed that under stimulation with the binary blend, both stimulus repetition rate (factor 1) and drug treatment (factor 2) were statistically significant (factor 1: *p *< 0.00001; factor 2: *p *< 0.01) in affecting the mean PFIs. The interaction between these two factors was also significant (*p *< 0.01), suggesting the extent of deterioration in tracking odor pulses was pulsing-rate dependent. Similar results were obtained from the single-component data. Together, these results indicate that: first, PN's pulse-following capability was significantly impaired by the actions of bicuculline; and second, although PNs generally improved their accuracy in tracking odor pulses that were delivered at a lower rate, the improvement was compromised under the influence of bicuculline. For example, under saline control, the PNs on average increased their pulse-tracking capability 7.4 times when the stimulus repetition rate dropped from 10 s^-1 ^to 0.2 s^-1^, but the improvement was only 2.3 times under bicuculline application (Figure 2c). We also discovered a striking difference between C-PNs (*n* = 4) and T-PNs (*n* = 6) in the way they resolved odor pulses (Figure 2d,e). Bicuculline significantly decreased the PFI values on T-PNs at 0.5, 1, and 2 s^-1 ^odor-repetition rates (two-way repeated-measures ANOVA at *p *< 0.05 level). The magnitude of reduction on each pulsing rate, however, was much higher in C-PNs, suggesting the C-PNs followed the odor pulses with higher contrast under control conditions. Nonetheless, application of bicuculline significantly impaired the pulse-following capability of both types of PNs.

The consistent bicuculline effect is best visualized in stacked autocorrelograms from all ten PNs, which reflect the underlying temporal structure of the responses to their specific ligands delivered at various repetition rates ranging from 0.2 to 10 s^-1 ^(Figure 2f,g). Under saline control, the collective autocorrelograms showed complete resolution of the repetitive odor pulses by these PNs up to 2 s^-1 ^(Figure 2f). In contrast, the same neurons started to lose odor-pulse tracking even at the rate of 1 s^-1 ^when bicuculline was applied (Figure 2g) and became worse at higher frequencies. The overall signal-to-noise ratio, in terms of representing odor pulses, was markedly lower when bicuculline was used. Similar results were obtained when the binary pheromone blend was used as odor stimulus.

To find out if other response features were altered by the application of bicuculline, we examined the averaged dose-response curves from 22 PNs (supplemental Figure 3 in Additional data file 1). The response magnitude was defined as the mean instantaneous firing rate within the response window (Materials and methods). In general, when the stimulus concentration was increased in decadal steps (0.1 to 100 ng/ml), the PNs' response magnitude also increased, regardless of whether a single pheromone component (C15 or Bal) or the binary blend (C15 + Bal) was used as stimulus. Moreover, the slope of the dose-response curve under bicuculline treatment was similar to that under the saline control, indicating that bicuculline did not alter PN's gain control mechanisms. Furthermore, the difference in response magnitude between the bicuculline treatment and the saline control was not statistically significant across the four odor concentration steps for all three bicuculline dosages – low (25 μM; *n* = 8; supplemental Figure 3a in Additional data file 1); intermediate (50 or 100 μM; *n* = 7; supplemental Figure 3b in Additional data file 1); and high (200 or 500 μM; *n* = 7; supplemental Figure 3c in Additional data file 1) – as analyzed by repeated-measures two-way ANOVA [26], *p *> 0.05. These results were in sharp contrast with those of pulse-tracking experiments, where the reduction of PFI values from the saline control due to the bicuculline treatment was statistically significant across a large range of odor-pulsing rates (Figure 2). In summary, these results demonstrated that bicuculline treatment significantly impaired PN's pulse-following capability but did not alter the detection and concentration coding of pheromone.

### Effects of bicuculline on odor-mediated flight behavior

**Figure 3:**
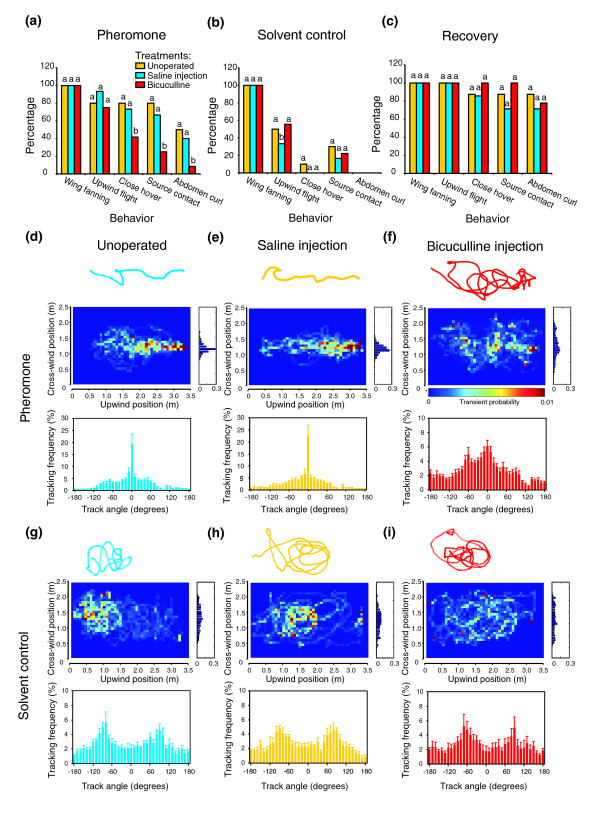
Bicuculline significantly affects pheromone-mediated navigation behavior. **(a-c) **Behavioral measurements on unoperated (gold), saline-injected (cyan) and bicuculline-injected (red) moths in a wind tunnel supplied with (a) pheromone or (b) solvent control (cyclohexane). Neither bicuculline nor saline injection affected a moth's ability to be motivated to fly (wing-fanning) or make upwind progress. A significantly lower percentage of bicuculline-injected moths (*n* = 12) displayed close hover, source contact and abdomen curl, compared with the unoperated (*n* = 10) and saline-injected (*n* = 15) groups (*G* test: *p *< 0.05). Under cyclohexane, all moths showed wing-fanning behavior, but only 30–50% of moths in each group (*n* = 10, 6, 9 for unoperated, saline-injected and bicuculline-injected, respectively) progressed upwind and an even lower percentage displayed close hover and source contact. None of the animals that came close to the source displayed abdomen curl. (c) The effects of bicuculline on close hover, source contact and abdomen curl shown in (a) were reversed after recovery for at least 2 h in a dark environmental chamber (*n* = 8, 7, 9 for unoperated, saline-injected and bicuculline-injected, respectively). Different letters within a behavioral category denote statistical significance (*G* test: *p* <0.05). **(d-i) **Flight-track analysis on unoperated (d, g), saline-injected (e, h) and bicuculline-injected (f, i) moths with pheromone or solvent control in the wind tunnel. (d, e) Using pheromone as the odor source, the unoperated and saline-injected moths flew directly toward the odor source, thus resulting in approximately straight flight tracks (top), centrally distributed transit probability (middle panels) and track-angle distribution histograms (bottom panels) with a prominent peak at zero degrees (mean ± SEM). The central distribution of transit probability is further demonstrated with a summed bar graph (along the wind direction) located to the right of the pseudocolor plots, showing a single peak at the center. (f) Bicuculline-injected moths, on the other hand, markedly diminished the central peak as well as the tracking frequency peak at zero degree track angle. (g-i) Replacing the pheromone with solvent control (cyclohexane) in the wind tunnel resulted in unanimous 'looping' flight tracks in all three treatment groups, reflecting an engagement of cross-wind casting in these moths, which is also shown in the randomly distributed transit probability of occupancy as well as in the bimodal distribution of track angle histograms.

Next we examined the relationship between the patterned activity of MGC-PNs and pheromone-modulated flight behavior. Bicuculline-injected, saline-injected, and unoperated moths were individually tested in a wind tunnel where the physicochemical conditions (air turbulence, pheromone emission rate) were dynamically scaled such that the estimated frequency of filaments within the odor plume was within the range of odor-pulsing frequencies where the bicuculline-induced reduction of PFIs was significant (Figure 2; supplemental Figure 4 and supplemental Table 1 in Additional data file 1). First, injections did not affect animals' ability to detect odor signal and fly upwind, as the injected and non-injected animals exhibited no statistical difference in wing fanning and upwind flight (*G* test: *p *> 0.05). Only 40% of the bicuculline-injected moths, however, hovered in front of the pheromone source, whereas nearly 80% of the unoperated and saline-injected moths did so, a difference that was statistically significant (Figure 3a; *G* test: *p *< 0.0001). Similarly, a significantly smaller fraction of the bicuculline-injected animals contacted the odor source (25% versus 80% for unoperated and 66.7% for saline-injected; *G* test: *p *< 0.0001) or displayed abdomen curling (8.3% versus 50% for unoperated and 40% for saline-injected; *G* test: *p *< 0.0001), which is a typical attribute of mating behavior (Figure 3a).

Next, to determine if the injections might have altered sensory processing of other stimuli such as visual and mechanical inputs, we performed behavioral tests similar to the experiments with pheromonal stimuli but using cyclohexane. Cyclohexane is not attractive to hawkmoths and thus serves as a negative control. Ten unoperated, six saline-injected, and nine drug-injected moths were tested under the same wind-tunnel conditions. About 55% of the bicuculline-injected moths flew upwind, which was not statistically different from that of unoperated and saline-injected treatment groups (50% and 33%, respectively; *G* test: *p *> 0.05). Among all these three groups only 20–30% of the animals contacted the solvent source. None of these moths showed the stereotypical close hovering and abdomen curling (Figure 3b). Furthermore, no significant difference was observed in flight speed between the injected (saline or bicuculline) and unoperated groups when presented either with cyclohexane or with pheromonal stimuli, although the flight speed toward cyclohexane was significantly higher than that towards pheromone (supplemental Table 2 in Additional data file 1). Bicuculline-induced changes in moth behavior were reversible. In another series of experiments, we allowed the moths to recover for at least 2 h after injections before testing them in the wind tunnel (*n* = 8, 7, 9 for unoperated, saline-injected, and drug-injected groups, respectively). The results showed that none of the behavioral measurements in the bicuculline group was significantly different from those of the other two control groups (Figure 3c). Interestingly, several behavioral parameters appeared to be improved compared with the moths without recovery (Figure 3a). This seems consistent with the observed enhancement of PFI after washing (Figure 2), suggesting that the recovered moths might have resolved odor filaments more effectively.

If the behavioral defects resulting from bicuculline injection were due to a disruption of the pulse-following capability of PNs, as shown in the physiological experiments (Figure 2), one would expect the flight tracks of the drug-injected moths to be different from those of the control animals. Indeed, the unoperated and saline-injected moths flew with more short upwind surges, resulting in significantly straighter tracks and higher flying speed than for bicuculline-injected moths (Figure 3d–f, flight tracks; supplemental Table 2 in Additional data file 1; one-way ANOVA: *p *< 0.001; *post hoc *Scheffé test: *p *< 0.01), which alternated more frequently between upwind surge and cross-wind casting. Similarly, the transit probability surface plots [27] demonstrated that the unoperated and saline-injected moths mostly occupied the central portion of the wind tunnel along the wind direction during flight whereas the bicuculline-injected moths flew more frequently across the wind direction, resulting in a more distributed transit probability density pattern (Figure 3d–f, pseudocolor plots). Analyzing the track angles of the flight trajectories of unoperated and saline-injected moths revealed a single peak at zero degrees, meaning that these animals spent more time heading directly toward the odor source. In contrast, the peak at zero degrees was severely diminished for the bicuculline-injected moths, suggesting that these animals could not maintain a flight course directly to the odor source (Figure 3d–f, histograms). When a pheromone source was replaced with a solvent control, the moths in all three groups (unoperated, saline-injected, bicuculline-injected) randomly flew over a large portion of the wind tunnel, as indicated by the transit probability plots (Figure 3g–i). Moreover, the track angle histograms of these animals showed bimodal distributions (Figure 3h,i), suggesting that the moths frequently engaged in cross-wind casting that is typically exhibited by unoperated moths searching for odor plumes.

**Figure 4:**
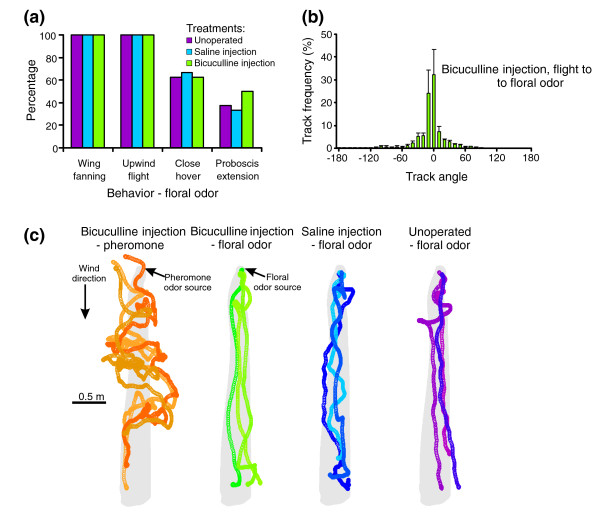
Injection of bicuculline into the MGC does not influence a moth's abilities to navigate to floral odors. **(a) **Behavioral measurements on unoperated (purple), saline-injected (blue) and bicuculline-injected (green) moths in a wind tunnel supplied with a floral odor. For all behaviors, there were no significant differences between treatments (*G* test: *p *> 0.10). *n* = 3–8 moths per treatment. **(b) **Measurement of track angles of bicuculline-injected moths flying toward floral odor source. A prominent peak at zero degrees indicates that the drug injected into MGC did not affect their navigation behavior mediated by floral odor. **(c) **Moth flight tracks to pheromone (orange) and floral odors (green, blue and violet). When injected into the MGC, bicuculline caused moths to increase the number of casts in the flight and a decrease in the ability to locate the pheromone source (orange flight tracks). In contrast, bicuculline injected into the MGC did not influence the ability of the moths to successfully navigate to, and locate, the floral odor source (green flight tracks). Saline-injected (blue flight tracks) and unoperated (violet flight tracks) moths exhibited similar flight behaviors to the floral odor as those moths treated with bicuculline. For each treatment three moth flight tracks were selected using a random number generator (denoted by tracks of different color shades). The tracks are made up of circles corresponding to video images captured at 0.016 s intervals.

To determine if the drug injected into the MGC could diffuse into other brain regions within the testing time frame that might affect the animal's odor-modulated behavior, in the final series of experiments we tested the responses of bicuculline-injected moths to floral odors in the wind tunnel. If attraction to the floral odors was significantly impeded, the drug injected into the MGC might have diffused and affected PNs elsewhere in the AL. The results of this experiment, however, did not support that possibility (Figure 4a). Like the unoperated (*n* = 8) and saline-injected moths (*n* = 3), 100% of the bicuculline-injected moths (*n* = 8) progressed upwind and hovered in front of the odor source, which was a white paper 'flower' loaded with a mixture of known, behaviorally effective floral volatiles that mimic the odor of an important floral food resource for *M. sexta *in southern Arizona [28]. In flight these moths moved more frequently toward the odor source, as reflected by the unimodal distribution of track angles (Figure 4b), resulting in relatively straight flight tracks (Figure 4c, floral odor tracks). About 60% of the moths in each group contacted the odor source, with no significant difference detected between the groups (*G* test: *p *> 0.05). Moreover, the percentage of moths in the bicuculline treatment and unoperated groups that extended their proboscis into the paper flower was not significantly different (50% and 37.5%, respectively; *G* test: *p *> 0.05). As a positive control, a few bicuculline-injected moths were flown to a pheromone source. They exhibited frequent alternation of upwind progression and cross-wind casting, confirming the disruptive effects of bicuculline on pheromone-plume tracking (Figure 4c, far left).

Taken together, all these findings support the hypothesis that bicuculline significantly affects moths' ability to orient to a pheromone source: that is, diminished zero-degree peak in track angle distribution histograms and a significantly lower percentage of moths displaying close hovering at the odor source, source contact, and abdomen curling. Bicuculline, however, did not affect their non-olfaction-mediated behaviors (for example, flying against wind, approaching a visual target and making turns, and so on). Moreover, the behavioral disruption was caused by effects of bicuculline within the MGC because the same drug treatment did not disrupt the orientation of moths to floral odors.

## Discussion

Searching for a particular pattern of neural activity responsible for a defined behavior is challenging because of the difficulty of establishing a causal link. In this study we confronted this problem by successfully disrupting MGC-PNs' ability to generate discrete bursts of action potentials and to follow repeated odor pulses that mimic the intermittency of natural odor plumes. Such a bursting response pattern was also observed in a previous study in which the moth was exposed to a pheromone plume and the electroantennogram (EAG) and firing activity of MGC-PNs were simultaneously recorded [21]. The discontinuous nature of wind-borne plumes was clearly demonstrated in that study by the individual EAG peaks that were found to be tightly correlated with the bursting responses of the PNs [21]. These findings suggest that MGC-PNs resolve the temporal discontinuity of a pheromone plume, which is known to be crucial for the flight behavior of a male moth seeking an unseen source of sex pheromone [6-10]. The bursts of spikes were locked to the haphazard, high-frequency contacts with pheromone filaments in the plume. A missing link, established in this study, was the causal relationship between the PNs' bursting response pattern and the odor-modulated flight behavior of the moth.

Bicuculline methiodide effectively and reversibly disrupted the ability of PNs to encode intermittent odor pulses (Figure 2), consistent with previous work, which also suggested that such disruption may result from antagonizing GABA_A _receptors in PNs [29,30]. This disruptive effect has now been more carefully quantified in the current study. The autocorrelation-based PFI was significantly lower for bicuculline-treated than untreated neurons for odor-delivery rates of up to 5 pulses s^-1 ^(Figure 2b,c), implying that the bicuculline treatment would affect the orientation behavior if a moth encountered odor filaments at frequencies of 5 pulses s^-1 ^or fewer in a natural plume. Through dynamic scaling of the turbulent conditions in our wind tunnel, we were able to control the filament frequency of the odor plume in the range of 1.98–2.5 pulses s^-1 ^as determined by EAG recordings, tracer plume experiments and anemometry (supplemental Figure 4 in Additional data file 1), and the estimated filament-encounter frequency was about 4 pulses s^-1 ^(Additional data file 1: experimental procedures and supplemental Table 1). Because of the boundary-layer effect around the moth antennae, which prolongs the pheromone concentration decay time [31], the ORC activation frequency may be further decreased from the encounter frequency, although biological and physical phenomena, including three-dimensional turbulence, kinematics of the moth flight (change in velocity, acceleration), and interaction between air movement generated by the moth wing-beat and the wind velocity [32,33], make accurate determination of the ORC activation frequencies difficult, if not impossible.

In our experiments, the flight-track analysis showed that although the unoperated and saline-injected animals spent most of the time heading directly toward the odor source, the bicuculline-injected moths were unsuccessful at steering a zero-degree track angle relative to the odor source despite being capable of making upwind progress (Figure 3d–f). As a consequence, a significantly lower percentage of bicuculline-injected moths exhibited close hovering, source contact, and abdomen curling (Figure 3a). These behavioral modifications are best explained by the alteration of PN response pattern caused by the action of bicuculline. Although clarifying the exact cellular mechanisms of bicuculline effects is beyond the scope of this study, our data suggest that these effects did not originate from the ORCs (supplemental Figure 5a–c in Additional data file 1) and were calcium dependent (supplemental Figure 5d–h in Additional data file 1).

According to a model proposed by Baker [11] based on studies of lepidopteran species, phasically modulated neural responses are responsible for generating upwind surges on contact with a pheromone plume, and separate tonic responses (resulting from non-olfactory input) are responsible for activating an internal counterturning program, the behavioral output of which is the cross-wind casting. Moreover, the tonic response can be inhibited by the odor-induced phasic response. Observations of *Drosophila melanogaster *differ noticeably from findings with moths in showing upwind surge even with a homogeneous odor cloud [27]. Our results, however, support the Baker model.

The bursting response generated by PNs upon contact with each odor filament is a critical component of the olfactory code responsible for upwind surges. In a natural odor plume, the arrival of odor packets at appropriate frequencies produces a series of fused upwind surges, which often appear as approximately straight flight tracks toward an odor source (Figure 3d,e). Transforming the discrete bursting response to prolonged excitation using bicuculline caused the moth to lose orientation toward the odor source and to perform the counterturning behavior more frequently (Figure 3f). The correlation between the prolonged excitation of PN response and the increased casting behavior suggests that this response pattern may function to shut down the upwind surge and unmask the internal tendency for casting. The internal counterturning program may be autonomously activated by non-olfactory stimuli at a center downstream from the AL, which may use a gating mechanism to filter the AL outputs carried by PNs. When there is no phasic (or bursting) input to this center, it may produce alternating antiphasic signals [34] that drive the casting behavior. The bursting responses of PNs, caused by intermittent stimulation, then inhibit the internal counterturning program, thus producing upwind surges. On the other hand, when the circuitry of this center is overloaded with PN inputs (prolonged excitation), it may become adapted and leave its alternating antiphasic output unmodulated. Behavioral experiments of moths in a homogeneous plume with unidirectional wind support this hypothesis [7,8]. In such an environment the animal receives long-lasting stimulation, which may cause heterogeneous response patterns among PNs. Some PNs may produce a continuous spiking response matching the stimulus duration [35], and others may produce random bursts within the stimulation period [29]. In either case the PN population as a whole may effectively cause their target neurons to adapt, resulting in casting behavior. Conversely, in nature the PN population may be entrained by stimulus dynamics, and thus only phasically activate their target neurons, resulting in upwind surge. Although bicuculline treatment altered the spontaneous spiking pattern of MGC-PNs (Figure 1; supplemental Figure 1 in Additional data file 1), these changes did not seem to affect the moth's crosswind casting behavior. Our data therefore suggest that the spontaneous firing pattern of MGC-PNs, whether or not modulated by drug treatment, contributes little if at all to the activation and sustaining of the counterturning program.

To determine the relationship between MGC-PNs' pulse-following ability and the pheromone-modulated orientation behavior of male moths, it is important to ask if the treatment with bicuculline also caused other changes, such as an altered firing rate, that might contribute to the moth's inability to track the odor plume in the wind tunnel. Experimental results showed, however, that the bicuculline treatment did not significantly change the response magnitude over a large range of pheromone concentrations (supplemental Figure 3 in Additional data file 1). Moreover, bicuculline treatment had no detectable effect on ORC activities whereas it did affect simultaneously recorded PNs (supplemental Figure 5a–c in Additional data file 1). This was probably due to some differences in ion conductances between ORCs and PNs. Thus, we conclude that the ability of PNs to respond to olfactory stimuli and encode odor concentrations (that is, to increase their firing rate proportionally to increasing odor concentration) are not affected by bicuculline treatment. Instead, the temporal response pattern is the feature that is significantly modified by the treatment.

Although bicuculline may not affect only the neurons associated with MGC, non-MGC neurons are unlikely to contribute to pheromone-mediated behaviors as pheromonal stimuli do not cross-excite non-MGC glomeruli [36,37]. Moreover, the experiments in which bicuculline was injected into the MGC of male moths that were subsequently tested for flight responses to behaviorally effective mixtures of floral odorants demonstrated that the drug-injected moths behaved as well as the unoperated animals in the wind tunnel (Figure 4). These findings suggest that little, if any, of the drug diffused beyond the MGC within the test time window, perhaps owing to the glial investment that ensheathes each glomerulus in the AL [38].

## Conclusion

On the basis of our findings, we conclude that the temporal pattern of MGC-PN responses (spiking bursts entrained to odor pulses), and not their magnitude (frequency of spiking), is significantly disrupted by injection of bicuculline into the MGC. The inability of moths to navigate successfully to and locate the pheromone source therefore most likely results from the loss of PNs' ability to track the individual filaments in an odor plume, rather than impaired detection and/or concentration coding of the pheromonal signal, and thus reveals a format of neural representation necessary for natural odor-seeking behavior.

## Materials and methods

### Preparation

*Manduca sexta *(L.) (Lepidoptera: Sphingidae) were reared in the laboratory on an artificial diet under a long-day photoperiod, and adult male moths, 4 days post-emergence, were prepared for experiments as described previously [23,39]. For electrophysiological recordings, the moth was restrained in a plastic tube with its head fully exposed. The labial palps, proboscis and cibarial musculature were then removed to allow access to the brain. To eliminate movement, the head was isolated and pinned to a wax-coated glass Petri dish with the ALs facing upward. Tracheae and a small part of the sheath overlying one AL were then removed with fine forceps. The preparation was continuously superfused with physiological saline solution containing 150 mM NaCl, 3 mM CaCl_2_, 3 mM KCl, 10 mM TES buffer pH 6.9, and 25 mM sucrose.

### Juxtacellular recording and dye-deposit technique

To allow long-term recording from single neurons, which is needed for the pharmacological experiments in this study, we used a juxtacellular recording and dye-deposition technique modified from [22]. In short, electrodes resembling those used for patch recording were pulled from thin-wall borosilicate glass capillaries using Sutter P-2000 laser puller and filled with a 4% solution of Lucifer Yellow CH (LY) (Sigma) in 0.2 M LiCl, resulting in <20 mΩ electrode resistance. The electrode shaft was filled with 0.1 M LiCl. An Axoprobe-1A amplifier connected to a 10× DC amplifier (Model FC-23B, WPI, Sarasota, FL) was used to amplify the signal up to 1,000×. Calibration pulses from the Axoprobe-1A amplifier were added to the output channels. A Leica micro-manipulator was used to advance the electrode into the MGC region of an AL until a contact similar to that used for perforated-patch recording was achieved. At this point, extracellularly recorded spikes could be distinguished from baseline noise. Recordings including activity from more than one neuron, as judged from spike amplitudes as well as the specificity of responses to pheromone components, were discarded. At the end of a recording period, the preparation was immersed in formaldehyde fixative solution (2.5% formaldehyde in 0.1 M sodium phosphate buffer containing 3% sucrose) with the electrode in place. The tip of the electrode was then quickly 'buzzed' to rupture the cell membrane, and current (-2 nA) was injected into the recorded neuron for 1–5 minutes. Because of the relatively large tip diameter of the patch-type electrodes used in these experiments, however, the 'impalement' by buzzing often resulted in current leakage and a low rate of success (<10%) of intracellular staining. Nonetheless, the injected fluorescent dye usually accumulated in the close vicinity of the electrode tip, forming a bright spot in the AL that clearly marked the glomerulus from which the recordings had been made.

### Sensory stimulation and characterization of neurons

Olfactory stimuli were delivered to the preparation by injecting odor-laden air puffs onto a constant air flow (1 liter per minute) that was directed at the middle of the antenna ipsilateral to the AL from which recordings were made. Trains of five air puffs (50 or 100 ms) with various inter-pulse intervals (5 s, 2 s, 1 s, 500 ms, 200 ms, 100 ms) were generated by means of a solenoid-activated valve controlled by an electronic stimulator (WPI). These air puffs were directed through a glass syringe containing a piece of filter paper, bearing various amounts of a single pheromone component (0.1–100 ng in decadal steps) or a blend of the same quantities of the two key pheromone components. The stimulus compounds used were: *E*10,*Z*12-hexadecadiennal (bombykal [Bal], the primary component of the conspecific female's sex pheromone) [25,40]; *E*11,*Z*13-pentadecadiennal ('C15', a chemically more stable mimic of another essential component of the sex pheromone) [24]; and a mixture of Bal and C15 (blend, 1:1 ratio). Although we substituted C15 for the natural pheromone component, we refer to both Bal and C15 as pheromone components.

MGC-PNs were characterized using three physiological criteria: randomly bursting spontaneous firing pattern; response specificity to pheromone components; and multiphasic pattern of responses. In *M. sexta*, uniglomerular MGC-PNs have been shown repeatedly to give predictable responses to the pheromone components according to the MGC glomerulus in which their dendrites arborize [35,39,41,42]: C-PNs are excited by antennal stimulation with C15 but inhibited (or not affected) by stimulation with Bal; T-PNs are excited by stimulation with Bal but inhibited (or not affected) by stimulation with C15; and both types of MGC-PNs are excited by the blend (Bal+C15). These types of PNs typically exhibit a triphasic (-/+/-) response pattern in intracellular recordings: that is, a brief inhibitory response (I_1_) preceding a depolarization phase that is then followed by a period of delayed after-hyperpolarization (I_2_). This characteristic pattern results from synaptic inputs from GABAergic, inhibitory local interneurons (LNs) as well as intrinsic properties of the PNs [42]. In juxtacellular recordings the brief I_1 _is difficult to detect; however, the silent I_2 _period is clearly visible. Finally, the spontaneous activity of MGC-PNs typically is more randomly bursting, whereas that of LNs is more tonic.

### Pharmacological manipulation

Bicuculline methiodide (Sigma-Aldrich, >95%) was diluted in physiological saline solution to different concentrations (25, 50, 100, 200, and 500 μM) and then bath-applied to moth preparations. A drip system comprising multiple 60-cc syringes converging on a central Teflon tube was used to facilitate quick switching from normal physiological saline solution to bicuculline solution and back. To minimize the disturbance to the physiological recordings, close attention was paid to the level of solution between syringes when switching from one syringe to another, ensuring an approximately constant rate of flow. The time when bicuculline took effect was determined by the change of spontaneous activity from randomly bursting to tonic. This time was about 10 minutes for concentrations of bicuculline >100 μM. To determine the role of extracellular Ca^2+ ^in inducing the bicuculline effects, we replaced the CaCl_2 _in the physiological saline solution with MgCl_2 _and then equalized the osmolarity with sucrose. Spontaneous activity and odor-evoked responses were first recorded under the normal physiological saline solution and then repeated under the Ca^2+^-free saline solution, bicuculline diluted in the Ca^2+^-free saline solution, bicuculline diluted in normal saline solution, and finally the normal saline wash. This series of treatments was designed to perform on a single MGC-PN.

### Simultaneous juxtacellular and sensillum tip recordings

To determine whether bicuculline-induced changes in MGC-PNs originate locally in the AL or from the periphery, sensillum tip recordings were performed simultaneously with the juxtacellular recordings from MGC-PNs. The antenna ipsilateral to the AL in which juxtacellular recordings were performed was gently twisted so that the long sensilla pointed upward. The tips of pheromone-sensitive type-I trichoid sensilla [24] were carefully clipped off with a pair of microscissors (Fine Science Tools, Foster City, CA) under a dissecting microscope; then a glass electrode filled with sensillum-lymph saline solution [43] was brought in contact with the cut end of a single sensillum using a Leica micromanipulator. As described earlier, the dual-channel Axoprobe-1A amplifer, a linear DC amplifier, and Datapack 2k2 system were used to achieve the simultaneous recordings.

### Data acquisition and analysis

Spike traces were digitized at 25 kHz sampling rate using Datapack 2k2 software (Run Technologies, Mission Viejo, CA), and the time stamp of each spike was extracted offline with the event-extraction function within the software package. The spike train data (columns of time stamps) were imported into a custom-written Matlab (The Mathworks Inc, Natick, MA) script, which first transformed the data column into a rate histogram at 5-ms bin width, and then calculated the autocorrelograms using the internal correlation function of Matlab. A simple PFI, which is based on the autocorrelograms, was calculated to reflect a PN's pulse-following ability. The peaks flanking the central peak on either side in the autocorrelograms are directly locked by odor pulses. Therefore,

$$PFI=C_{ipi}/\left(\sum_{lag=0}^{ipi}C/n\right)$$

where *ipi *is the inter-pulse-interval (5 s for 0.2 Hz, 2 s for 0.5 Hz, and so on), *C*_*ipi *_is the correlation value at the specified ipi derived from correlograms, *C *is a vector of correlation values between the central peak (that is, lag = 0) and the peak at *ipi*, and *n *represents the number of time bins between these two peaks. This ratio index essentially reflects the contrast of spike density between the bursting response driven by odor pulses and the period between two adjacent bursts. The better a neuron resolves odor pulses, the higher the PFI value is.

To determine the width of the response window, the spike train data were exported into Neuroexplorer (Nex Technologies, Littleton, MA) for plotting the peristimulus time histograms, which allowed approximate estimation of response duration. Then the average of instantaneous spiking frequency (that is, the inverse of inter-spike interval) within the response window was calculated using a custom-written Matlab script for each odor-evoked spike burst, and finally these averages from all five trials (pulses) were averaged again to obtain the grand average. The prolonged excitatory responses caused by the bicuculline application were cut off at the 250 ms window, in which most of the odor-evoked responses under physiological saline condition fell. The measurement of response magnitude, defined as the grand average of instantaneous spiking frequency, is robust to the variations in actual response durations. We compared the dose-response curves calculated using a 250 ms window with that using a 500 ms window and did not observe significant differences. All statistical comparisons were performed using the Statistics Toolbox of Matlab or a third-party program downloaded from the Matlab website [26].

### Microinjection

A 4-day-old moth was restrained in a plastic tube 60-90 minutes prior to scotophase and kept at room temperature in the light awaiting surgery and injection. Moths were de-scaled entirely from the nape of the neck to the labial palps. A rectangular window was cut in the head capsule, horizontally above the nape of the neck, extending the length between the antennae and short of the labial palps. The window was removed and pushed forward, keeping the connective tissues and muscles attached. The MGC regions in both ALs were located by gently pushing muscle fibers and connective tissues aside with fine forceps and then were injected with 500 μM bicuculline or physiological saline solution. Injection was accomplished via Quartz pipettes (OD 1.0 mm, ID 0.70 mm, Sutter Instruments, San Diego, CA) pulled with a Model P-2000 laser puller (Sutter Instruments) using the same program for pulling intracellular electrodes. Pipettes were filled with the solution to be injected and connected with an output line of a dual-channel Picopritzer (General Valve Corp, East Hanover, NJ). The pipettes were then clipped at the tip with fine forceps to allow solution passage. Pipettes were manually inserted into the MGC in each AL and 10 drops (mean diameter ± SD: 76 ± 9.2 μm) were administered quickly in succession with a step pedal that controls the Picospritzer. After injection, the cuticle window was repositioned and sealed with myristic acid (Sigma), and the moth was removed from the plastic tube and placed in an individual cage to recuperate under the same conditions in which it had eclosed. Post-surgery moths were taken 20-30 minutes into scotophase for flying in the wind tunnel. To see whether moths could recover from bicuculline injection, these animals were kept in the dark for at least 2 h before testing them in the wind tunnel.

### Wind-tunnel experiments and data analysis

A Plexiglas wind tunnel (L × W × H = 4 × 1.5 × 1.5 m) was used to create a highly controlled wind-flow environment for examining upwind flight behavior in response to pheromone plumes. The wind-tunnel conditions were physicochemically scaled to match the odor emission rate equivalent to that of one female moth and the filament frequencies used in physiological experiments (supplemental experimental procedures in Additional data file 1). Longitudinal (u) wind speeds in experiments were 20 cm/s. At the beginning of scotophase, naive, adult male moths were placed individually 3.5 m downwind from the odor source. Each moth was allowed to fly freely inside the wind tunnel for 5 minutes, during which its behavior was recorded. Two types of behavioral data were acquired during experiments: video acquisition and subsequent motion analysis of moth flight behavior for each treatment group (see supplemental experimental procedures in Additional data file 1 for details); and scoring of moth behaviors. Scored behaviors were: wing fanning (typical behavior just prior to flight), upwind flight (moth comes within 0.75 m of odor source), close hover (moth hovered within 10 cm of odor source), source contact, and abdomen curl (a typical mating posture).

Experimental treatment groups included three drug treatments and two odor stimuli, thereby creating a 3 × 2 experimental series. Treatments were tested in a randomized-block design where each block contained positive and negative controls for stimuli and drug treatments. Drug treatments were unoperated moths (to control for surgery effects), saline-injected moths (to control for injection effects), and bicuculline-injected moths. Moths were flown individually to either of two odor stimuli: the two-component pheromone blend or the cyclohexane (negative) control. Four microliters of 500 ng/μl of the pheromone blend (2 μg total), or 4 μl of cyclohexane, were pipetted onto a filter paper placed in the upwind portion of the wind tunnel. This pheromone concentration closely mimicked pheromone emission rates of calling females (supplemental Table 3 in Additional data file 1; see supplemental Figure 6 and experimental procedures in Additional data file for details on pheromone headspace collection and GCMS analysis). For the pheromone odor stimuli, 15, 10, and 12 moths were used for the unoperated, saline-injected, and bicuculline-injected treatment groups, respectively. For the cyclohexane (control), *n* = 10, 6, and 9 moths for the unoperated, saline-injected, and bicuculline-injected treatment groups, respectively.

Two more series of control experiments were conducted to determine the duration of the drug effect and the approximate diffusion range. To determine if moths recovered their ability to track the odor plumes after injection, a similar injection protocol, drug-treatment group (un-operated, saline-injected, bicuculline-injected), and pheromone stimulus were used, but this experimental series differed from the previous one in one important aspect; moths were flown 2-3 h post-injection. For these experiments, 8, 7, and 9 moths were used for the un-operated, saline-injected, drug-injected treatment groups, respectively. A last experimental series examined whether bicuculline injected into the MGC diffused into neighboring regions of the olfactory system devoted to food odors and impaired behavior to those odors. Three treatment groups (unoperated, bicuculline-injected, and saline-injected; *n* = 8, 8, and 3, respectively) were flown to floral odors.

The scored categorical variables 'wing fanning', 'upwind flight', 'close hover', 'source contact', and 'abdomen curl' were analyzed by means of a log-likelihood test (*G*-test) when testing overall treatment effects and when comparing pairs of proportions. An α-level of significance of 0.05 was used. The digitized flight-track analyses (flight speed, acceleration, heading angles) were analyzed using one-way analysis of variance (ANOVA) because data met the assumptions of this test.

## Authors' contributions

SLG performed drug injection experiments. JAR designed, performed and analyzed data from wind-tunnel experiments. HL designed, performed and analyzed data from electrophysiological experiments, and participated in wind-tunnel experiments. HL, JAR and JGH together wrote the manuscript.

## Additional data files

Additional data file 1  includes additional experimental procedures and additional figures and tables. Arizona Research Labs Division of Neurobiology (ARLDN) wind tunnel and Video Acquisition and Motion Analysis System (VAMAS); physicochemical scaling of the wind tunnel conditions; supplemental Table 1, the relationship between wind velocity, distance between odor filaments and the moth's airspeed of flight; supplemental Table 2, moth flight speeds (x-, longitudinal-axis) as a function of odor stimulus and treatment; supplemental Table 3, scaling of synthetic pheromone emission rates to those of female *M. sexta*; supplemental Figure 1, bicuculline effect on spontaneous firing pattern; supplemental Figure 2, bicuculline effect on response pattern of PNs; supplemental Figure 3, MGC-PN's response magnitude was not significantly affected by bicuculline treatment; supplemental Figure 4, anemometry, EAG, and tracer-test results demonstrating plume turbulence and filaments; supplemental Figure 5, bicuculline effects on MGC-PNs are not originated from the olfactory receptor neurons and are calcium dependent; supplemental Figure 6, analytical GCMS comparison of the natural pheromone and a synthetic standard of the two pheromone components, (E,Z)-10,12-hexadecadienal (Bal) and (E,E,Z)-10,12,14-hexadecatrienal (EEZ).

## Supplementary Material

Additional file 1 Additional experimental procedures and additional figures and tables Click here for file
