# Supplementary material for: Contrast enhancement of stimulus intermittency in a primary olfactory network and its behavioral significance
Source: J Biol. 2009 Feb 20;8(2):21. doi: 10.1186/jbiol120 (PMC2687775; doi:10.1186/jbiol120)
Supplement: Additional file 1 — Additional experimental procedures and additional figures and tables [file jbiol120-S1.doc]

**Contrast enhancement of stimulus intermittency in a primary olfactory network and its behavioral significance**

Hong Lei, Jeffrey A. Riffell, Stephanie L. Gage and John G. Hildebrand

**Supplemental Experimental Procedures**

##### Arizona Research Labs Division of Neurobiology (ARLDN) wind tunnel and Video Acquisition and Motion Analysis System

##### The ARLDN Plexiglas ® wind tunnel (L x W x H = 4 x 1.5 x 1.5 m) creates a highly controlled unidirectional flow environment for creating and measuring semiochemical plumes. The wind tunnel functions by forcing air into the upwind end of the tunnel through a carbon filter and multiple screens to produce unidirectional flow, and exhausted at the downwind end through a duct vented into a laboratory fume hood. The wind speed was set at 20 cm/s. Prior to each trial, 4 μl of 500 ng/μl of the 2:1 pheromone blend (2 μg total) (pheromone stimulus) or 4 μl of cyclohexane (control stimulus) was pipetted onto a filter paper placed in the upwind section of the tunnel directly in the middle of the tunnel. Red and white light (0.5 lux) provided the light necessary for video recording of insect flight behavior. Video of moth flight behavior was recorded overhead through a CCD video camera (Cohu model 6415-2000) with a macro lens. Images were captured on analog tape and converted to digital format and processed with a Peak Motus Corp. (Peak 3D, v7.2) video acquisition and video motion analysis system interfaced to a WindowsXP computer. Digitized moth flight tracks were analyzed for speed, instantaneous track acceleration, and orientation (track angle) relative to the odor source defined as the 0° origin. Transit probability surface plots, derived from superimposed flight trajectories, were used to show the variation of flight tracks [27]. Transit plots were calculated by dividing two-dimensional views of the wind tunnel into 1367 squares with side lengths of 8 cm. For each treatment, the number of moth occurrence within each square was summed and divided by the total number of occurrences in all squares to yield a probability of square occupancy, where the total probability summed to 1.0. For each treatment, the cross-wind distribution of moth occupancy is shown by the histogram on the right of each surface plot.

***Physicochemical scaling of the WT conditions***

***Pheromone collection, characterization, and quantification of emission rates.*** *M. sexta* pheromones were collected from virgin 2-day old females using dynamic headspace sorption. *M. sexta* pheromones have previously been characterized by LCMS [40]. In that study, pheromones were collected through gland extracts. In the present study, we wished to make our own absolute determinations using dynamic headspace sorption to scale our synthetic pheromone emission rates to that of an actively calling female *M. sexta*. Virgin females were enclosed in a 3 L glass jar with a removable top with holes for air exchange. Charcoal-filtered air was pumped into the jar at 2 L/min, and odor-laden air was withdrawn by vacuum at 2 L/min. The vacuum line was used to pull headspace air through sorbent cartridge traps constructed by packing 100 mg of Super Q adsorbent (mesh size 80–100) into borosilicate glass tubes (7 mm) plugged with I.D.#4 silanized glass wool. Pheromone collections began at anthesis (near sunset for all moths) and continued overnight for up to 12 hours. Eight collection replicates were collected from eight female *M. sexta*.

***Odor analysis.*** Trapped volatiles were eluted from sorbent cartridges using 600 μL of HPLC grade hexane. The sample was concentrated down to 15 μl by gently blowing N_2_ gas to remove the hexane. Each sample was stored in 2 mL borosilicate glass vials with Teflon-lined caps at -80 °C until analysis. 1 μL of the volatile sample was injected into and analyzed with a GC–time-of-flight mass spectrometry (TOF-MS) system consisting of an HP 6890 (Agilent Technologies, Palo Alto, CA, USA) gas chromatograph and a Waters TOF-MS (Waters-Micromass, Millford, MA, USA) used in electron impact ionization mode at 70 eV. Two GC columns (J&W Scientific (Folsom, CA) were used: a DB1 (30 m, 0.25 mm, 0.25 µm) and a DB5 (30 m, 0.25 mm, 0.25 µm). Helium was used as a carrier gas at constant flow of 1 ml/m. The initial oven temperature was 55° C for 7 min followed by a heating ramp of 15° C per min until 160° C, which was held isothermally for 2 min. A second heating ramp increased at 6° C per min until 250° C, and was then held isothermally for 4 min. Mass chromatogram peaks were initially identified through use of the NIST mass spectral library of ca. 130,000 mass spectra and verified by injection of authentic standards (generously provided by J. Millar, UC Riverside). Peak areas for each compound were integrated using MicroMass MassLynx software and are presented in terms of relative abundance as percent of total fragrance emitted. Pheromone peak areas were quantified using the internal standards and expressed in units of nanograms per female per hour.

***Turbulent wind measurements.*** A challenging task in almost all studies of insect navigation is to characterize the physical conditions within the WT (but see [44]). Although baffles are commonly used to induce turbulence, the style and type of baffle varies from study to study. Since little information beyond the mean wind speed are provided, reproducing similar physical conditions – and hence, similar odor dynamics – is unlikely. In contrast to research in insect behavior, wind-tunnel experiments by fluid engineers have provided a reproducible manner by which to control turbulent flow: through grid- and baffle-generated turbulence. Aerodynamic characteristics of induced turbulence are well known for producing near isotropic turbulence such that, for a given baffle size (*D*) and mean wind velocity (*ū*), the turbulent eddy frequency (*n*) and distance (*L*) can be predicted. The non-dimensional Strouhal number (*S_t_*), indicates the frequencies by which eddies are shed from physical objects, and is calculated by *S_t_ =* (*nD*/*ū*). At higher Reynolds numbers (i.e., the ratio of inertial to viscous forces) equivalent to the turbulent environment which *M. sexta* experiences (Re = 10^2^ - 10^3^), the Strouhal number remains approximately 0.2. Thus, the eddy frequency within a WT can be determined by *n* = (0.2*ū* /*D*), and eddy distance by *L*= *ū* /*n*. By placing the odor source flush to the downwind surface of a baffle, odor dynamics can be dictated by eddies (now odor-laden filaments) shed from the baffle, rather than the net airflow. Although the Strouhal number only approximates the turbulent eddies being shed from the baffle, it provides a first-ordered approach towards manipulation of the turbulent levels in the WT. Here, the WT wind velocity (20 cm/s) and baffle diameter (3 cm) were used to create a filament frequency of 1.3/s to examine its effects on *M. sexta* navigational behavior.

The scale of turbulence in the tunnel was assessed using a 3-D sonic anemometer (Young81000, R.M. Young Co., Traverse City, Michigan, USA). This instrument has a resolution of 1 mm/s root mean square (rms). Orthogonal wind components *u*, *v*, and *w*, where *u* is the instantaneous streamwise velocity measured along the wind tunnel, *v* is the instantaneous lateral component of velocity parallel to the wind tunnel floor, and *w* is the instantaneous vertical component of velocity, were sampled at 32 Hz at four positions along the tunnel’s centerline ca. 60 cm above the floor (the height of the plume during anemometer, smoke, and EAG measurements). Digital outputs were recorded on a laptop computer. Mean velocities, turbulent intensities of velocity fluctuations (calculated as the square root of the mean of the squared fluctuation about the mean velocity in the case of the streamwise component, or the square root of the mean of the squared values of the fluctuating velocities in the case of the cross-flow components, and referenced to the streamwise mean), and Reynolds stresses, a measure of the turbulence fluctuations in the wind tunnel, is calculated by:

, (1)

where the term is the mean of the crossproduct of fluctuating velocities in the longitudinal (*u*) and vertical (*w*) dimensions and the negative term in front of the air density (*ρ*) denotes the downward flux of momentum. In addition, the crossproduct of fluctuating velocities in the transverse (*v*) and longitudinal (*u*) dimensions, and the transverse (*v*) and vertical (*w*), dimensions were determined.

***Tracer smoke test.*** To visualize the turbulent odor plume within the wind-tunnel, 100 μl of TiCl_4_ was pipetted onto the filter paper placed in the upwind section of the wind tunnel that was located in the same position as the pheromone source. Vaporization of the TiCl_4_ created a tracer plume that mimics the plume dynamics of a pheromone plume. The TiCl_4_ plume was illuminated with a narrow (ca. 2 cm thick) light sheet emitted from a halogen lamp that was connected to a collimator which focuses the light into a sheet (ca. 2 cm thickness and 20 cm width). The tracer plume was videotaped at four locations downwind from the source (25, 50, 100, and 300 cm) for 20 s at 30 frames/s with a CCD video camera (Cohu model 6415-2000) with a macro lens. The analog video was digitized and the smoke plume analyzed through ImageJ software where plume intensity was measured as a function of grayscale intensity with the “Intensity vs. Time” algorithm from a “region of interest” which was 2 mm^2^ in the plume centerline. The analysis thereby created a 600 frame time series of the plume intensities at each downwind location. Each data set was analyzed for “filament frequency” defined as the frequency at which grayscale intensity was above the noise threshold and spectral analysis of the time series using Matlab (v. 7.0.4; Natick, MA USA) where the analysis applies a Fast Fourier Transforms algorithm to convert the time series into a frequency spectrum that can be plotted as a periodogram.

***EAG wind tunnel recordings.*** The electroantennogram (EAG) probe (PRG-2, Syntech) was mounted on a stand inside the wind tunnel in the plume centerline as determined from tracer plume experiments. Antennae of *M. sexta* produced little or no mechanoreceptor response to turbulent wind fluctuations of clean air (Supplemental Fig. 2 A), and showed no periodocity. To inhibit electrical interference, the stand was grounded to a layer of aluminum foil that covered the inner surface of the wind tunnel 50 cm upwind and downwind of the EAG probe. Males 2–3 days old were placed in a plastic tube with it’s antenna mounted between the recording and reference electrodes using electrode gel (Spectra 360, Parker Laboratories Inc., NJ, USA) with the ventral side of the antenna facing upwind. EAG signals were acquired through a PC interface board (IDAC-02, Syntech) and Autospike32 (Syntech) software. The frequency of EAG deflections, defined as 50% return to baseline calculated using the peak amplitude and depolarization value at the onset of the following peak [45], was determined for each 100 s recording period at each position (25, 50 100, and 300 cm from pheromone source) in the wind tunnel. Data was exported and analyzed for mean deflection frequency at each position for each male (*n* = 6) and spectral analysis of the EAG time series using Matlab (v. 7.0.4; Natick, MA USA) where the analysis applies a Fast Fourier Transforms algorithm to convert the time series into a frequency spectrum that can be plotted as a periodogram.

**Supplemental Table 1.** The relationship between wind velocity, distance between odor filaments and the moth's airspeed of flight. Interactions between these variables determine a moth's realized frequency of odor-filament encounter. Realized encounter frequency = Realized moth flight speed / (Wind-tunnel air speed / Filament frequency). The ORC activation frequency may differ from the calculated encounter frequency due to boundary layer effects around the moth antennae which may act non-linearly on the decay time of the stimulus around the ORCs [31].

|  | WT distance from odor source (cm) | | | |
| --- | --- | --- | --- | --- |
|  | 25 | 50 | 100 | 300 |
|  |  |  |  |  |
| Wind-tunnel air speed (cm/s) | 16.97 | 19.92 | 19.31 | 19.75 |
|  |  |  |  |  |
| Filament frequency (#/s) | 1.98 | 2.03 | 1.98 | 2.50 |
|  |  |  |  |  |
| Filament distance (cm) | 8.56 | 9.80 | 9.74 | 7.90 |
|  |  |  |  |  |
| Realized moth flight speed (cm/s)* | 32.57 | 35.52 | 34.91 | 35.35 |
|  |  |  |  |  |
| Realized encounter frequency (#/s) | 3.80 | 3.62 | 3.58 | 4.47 |
|  |  |  |  |  |

* Speed along the *x*-axis (wind direction).

**Supplemental Table 2**. Moth flight speeds (*x*-, longitudinal-axis) as a function of odor stimulus and treatment.

| Stimulus | Unoperated (control) | Saline injection (control) | Bicuculline injection |
| --- | --- | --- | --- |
|  |  |  |  |
| Pheromone | 15.30 (1.69) | 16.78 (1.78) | 13.43 (2.07)* |
|  |  |  |  |
| Cyclohexane | 58.62 (4.02) | 66.38 (5.50) | 68.93 (5.98) |
|  |  |  |  |

Values are means ± s.e.m. (N>7 for each treatment).

*P<0.05; an asterisk denotes significantly difference between treatments in the stimulus factor (one-way ANOVA with post hoc Scheffé test).

**Supplemental Table 3**. Scaling of synthetic pheromone emission rates to those of female *M. sexta*. During scotophase a calling female moth emits two major sex pheromone compounds in addition to six others that have been previously shown to mediate male anemotactic and copulatory behaviors: (*E,Z*)-10,12-hexadecadienal (Bal) and (*E,E,Z*)-10,12,14-hexadecatrienal (EEZ). From dynamic headspace sorption and GCMS analysis we found these two pheromone components to be at a ca. 2:1 ratio and a total emission rate of 3.09 ng/h/female (± 0.714 SEM), similar to results from Tumlinson and colleagues [25,40]. Dynamic headspace sorption and analysis of the synthetic pheromones at the concentration used in behavioral experiments, (*E,Z*)-10,12-hexadecadienal (Bal) and (*E,Z*)-ll,13-pentadecadienal (C15) (a close mimic of EEZ), revealed similar total emission rates (2.672 ng/h, ± 0.491 SEM) that were not significantly different from the female moth emissions (one-way ANOVA: *F*_1,38_ = 0.13, *P* = 0.71). Moreover, the emission rate of Bal was not significantly different between the female and the filter paper loaded with the pheromone (one-way ANOVA: *F*_1,18_ = 0.50, *P* = 0.48). The synthetic pheromone components (Bal and C15) were set at a 1:1 ratio because of the lower efficacy of the C15 component in relation to EEZ (5).

|  | | | | |
| --- | --- | --- | --- | --- |
| Pheromone odorants |  | Pheromone emissions (ng/h) | | |
|  |  | Female *M. sexta* (n=12) |  | Synthetic pheromone (n=8) |
|  |  |  |  |  |
| (*E,Z*)-10,12-hexadecadienal |  | 1.965 (0.770) |  | 1.256 (0.395) |
|  |  |  |  |  |
| (*E,E,Z*)-10,12,14-hexadecatrienal |  | 1.131 (0.295) |  | NA |
|  |  |  |  |  |
| (*E,Z*)-ll,13-pentadecadienal |  | NA |  | 1.416 (0.600) |
|  |  |  |  |  |
|  |  |  |  |  |

**Supplemental Figure 1**

**
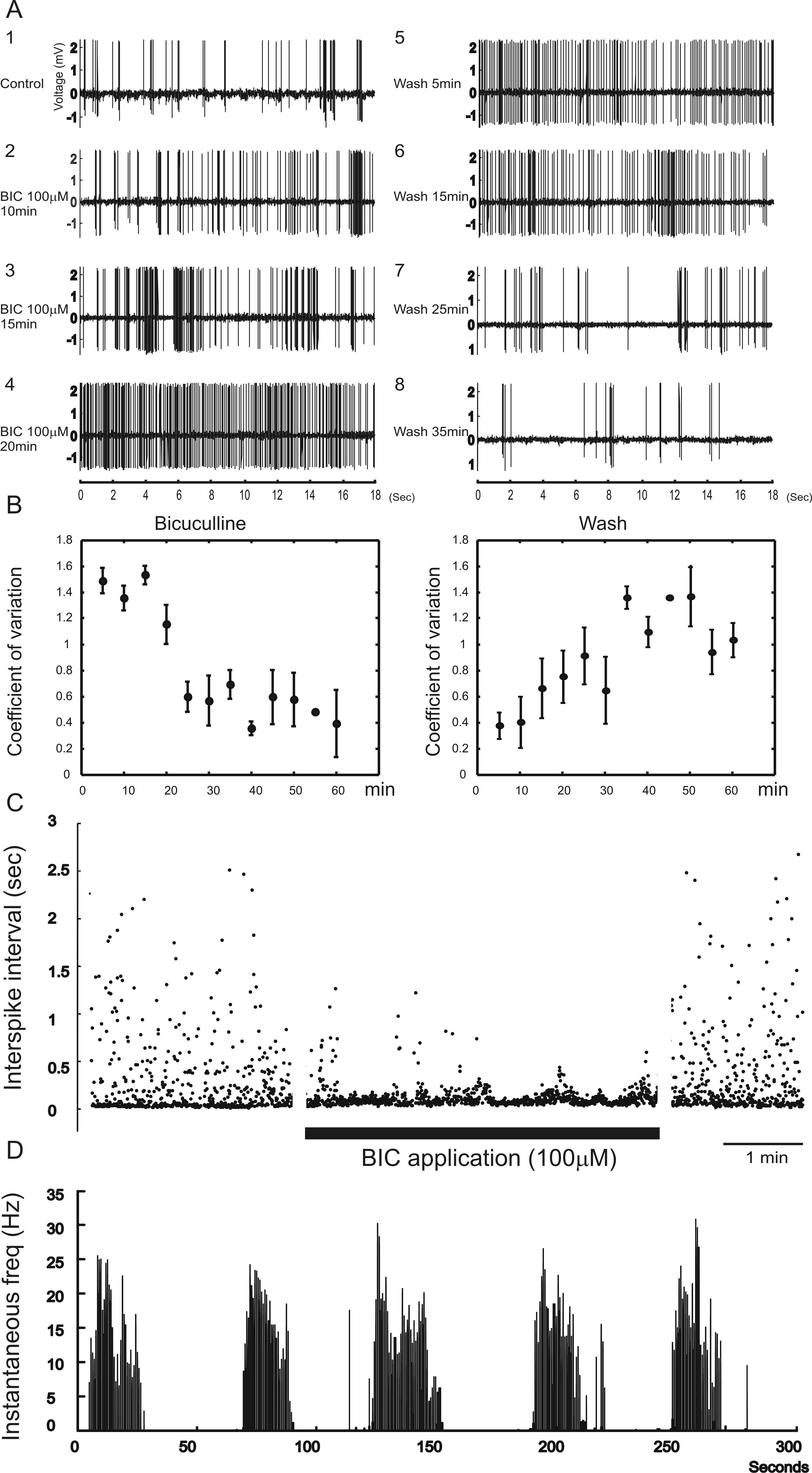
**

**Supplemental figure 1 Bicuculline (BIC) effect on spontaneous firing pattern**

(A) Shown with raw juxtacellular spike traces, bath application of 100 μM bicuculline reversibly changed the spontaneous firing pattern of an MGC PN from random bursting to tonic pattern (1-8).

(B) Coefficient of variation was calculated for n=10 PNs to reflect the evolving changes of spontaneous firing pattern under bicuculline and saline wash conditions.

(C) Consequently, the interspike interval during the drug effective period was less variable than that during the periods before and after the drug application. The black bar indicates the drug treatment period. Notice the gaps preceding and following the drug treatment period, which indicate the three data sections were not continuous in time.

(D) Bicuculline treatment also resulted in a periodic appearance of tonic firing pattern, shown in an instantaneous rate histogram over a 5 min period.

**Supplemental Figure 2**

**
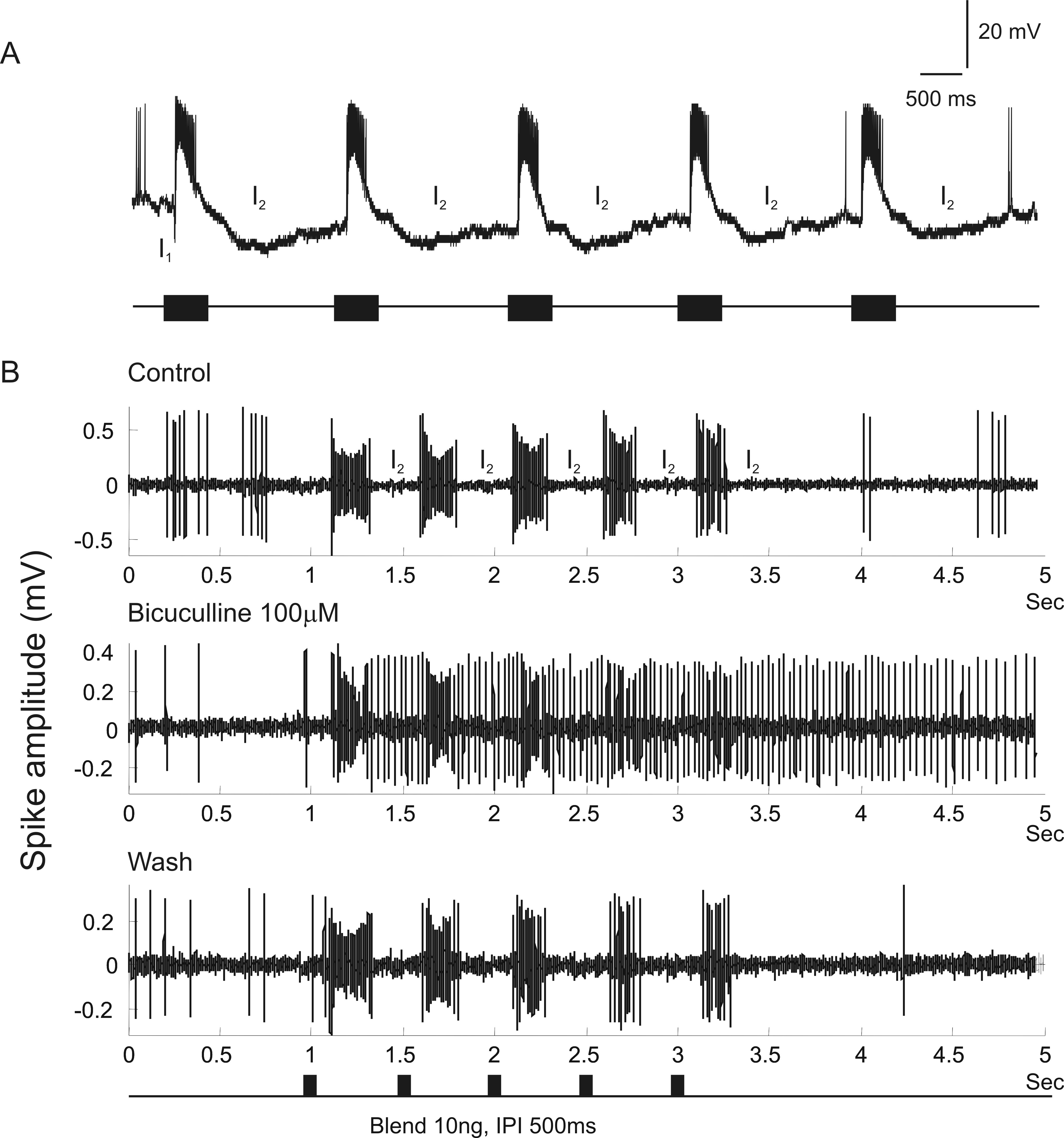
**

**Supplemental figure 2 Bicuculline effect on PN’s response pattern**

(A) Intracellular trace showing the typical response pattern of an MGC-PN to five odor pulses (blend 10ng). In response to the first pulse the PN produced a brief inhibitory postsynaptic potential (I_1_), which was then followed by a strong excitatory phase that was sharply terminated by the second inhibitory potential (I_2_). In remaining responses the I_1_ was not obvious, the I_2_, however, followed every single excitatory phase.

(B) Juxtacellular recordings from a different MGC-PN demonstrating the bicuculline (100μM) effect on PN’s response pattern. Under saline control the PN produced 5 excitatory responses locking onto each of the 5 odor pulses (blend 10ng). All 5 responses were apparently followed by silent I_2_ periods. Bicuculline application completely blocked the I_2_, resulting in prolonged excitatory responses. This effect was reversed by saline wash. Black bars under traces indicate odor pulses.

**Supplemental Figure 3**

**
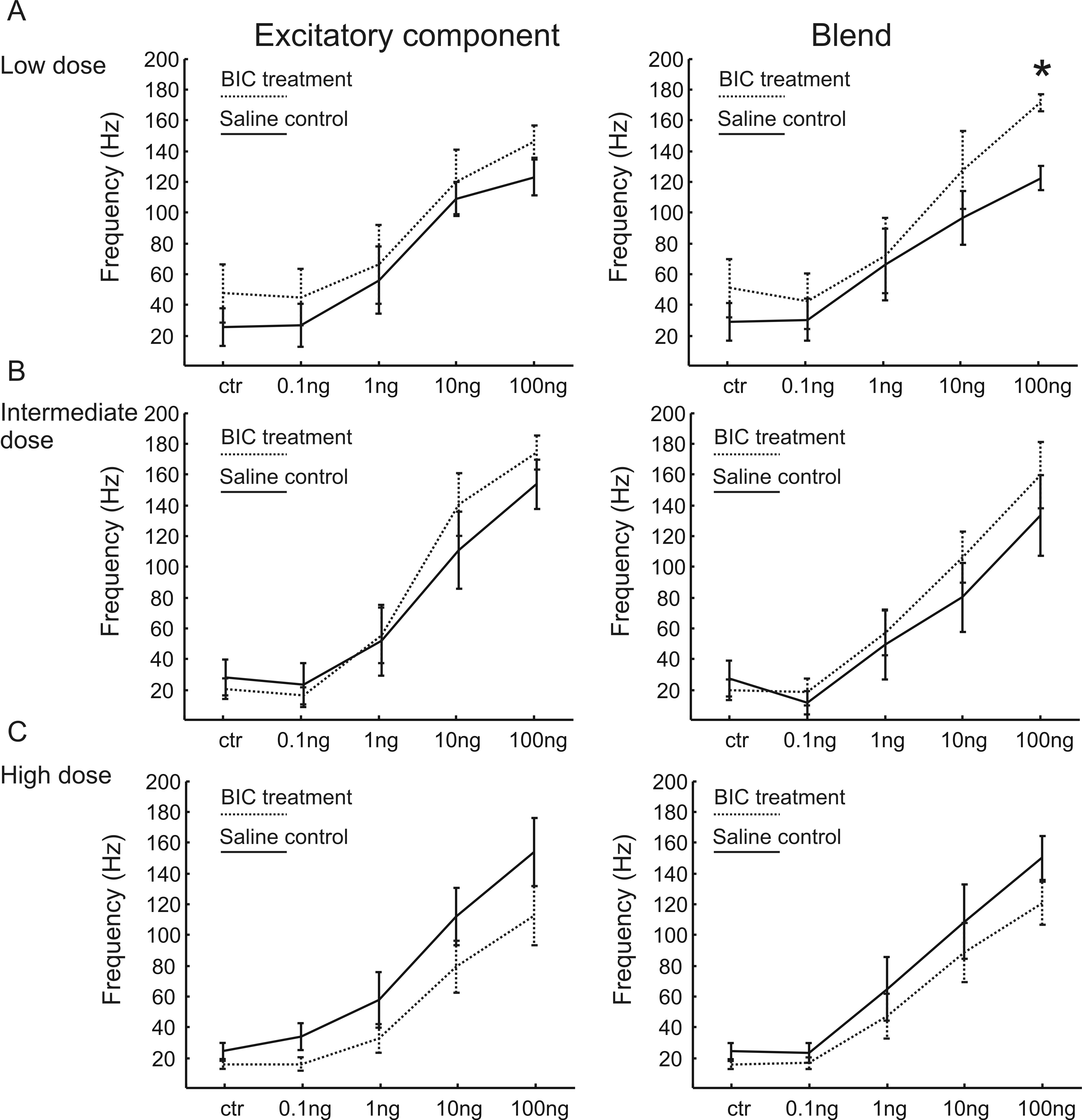
**

**Supplemental figure 3. MGC-PN’s response magnitude was not significantly affected by bicuculline (bic) treatment**

(A) Dose-response curves (mean ± S.E.M., n=8) across 4 orders of magnitude in stimulus concentration showing non-significant difference between saline control (solid line) and low-dose (25 μM) bicuculline treatment (dotted line) (Repeated-Measure Two-Way ANOVA). Similar results were obtained for using the single excitatory pheromone component (left panel) or the blend (right panel) as stimulus except at the highest odor concentration (Asterisk indicates statistical significance).

(B) At intermediate dosages (50 μM or 100 μM, n=7) and (C) high dosages (200 μM or 500 μM, n=7) bicuculline treatment did not cause significant changes (dotted line) in response magnitude to excitatory pheromone component (left panel) or the blend (right panel) across the 4 log steps of odor concentrations, comparing with the saline control (solid line).

**Supplemental Figure 4**

**
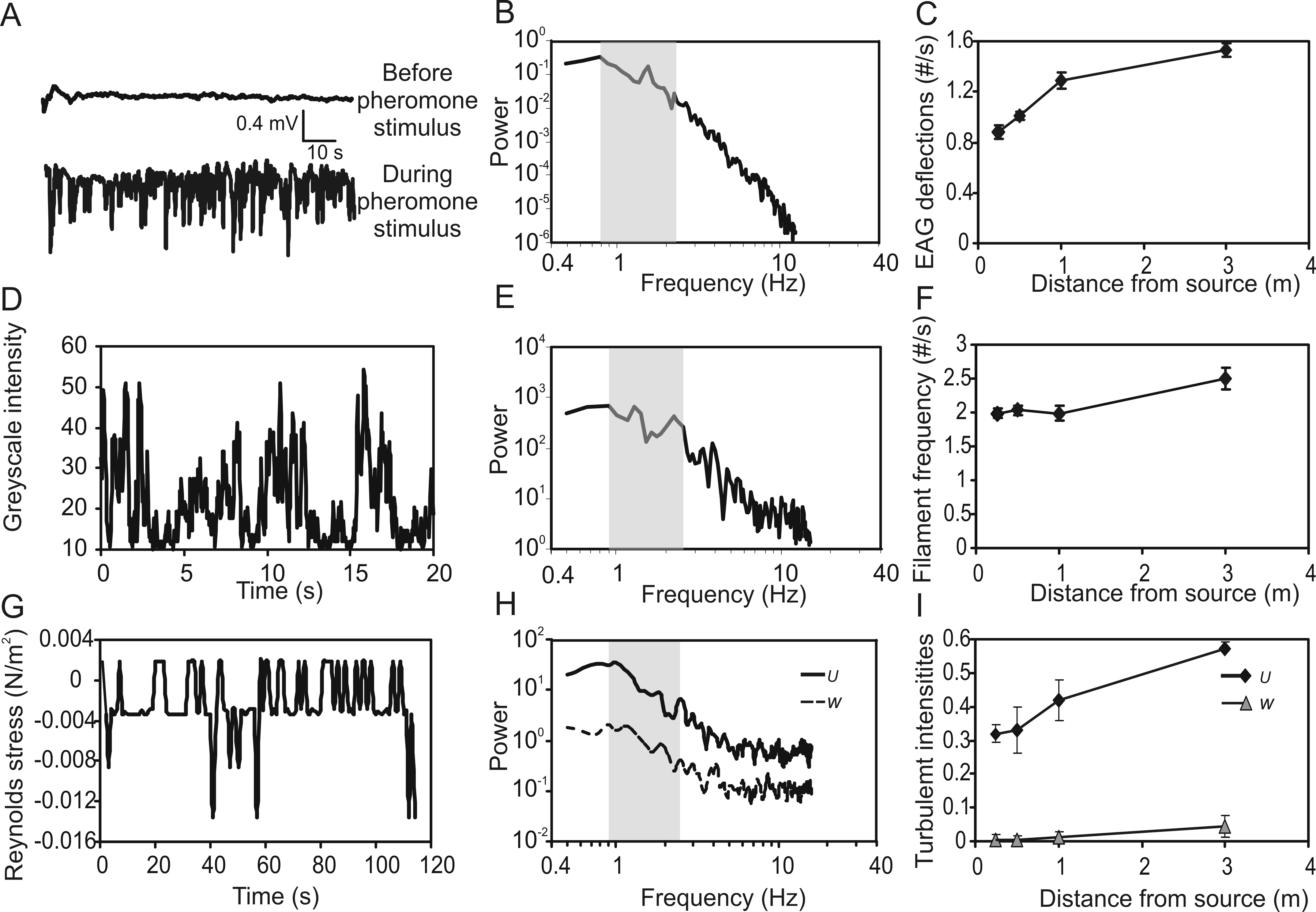
**

**Supplemental figure 4. Anemometry, EAG, and tracer-test results demonstrating plume turbulence and filaments*.*** First, EAG recordings (*n* = 6) indicate that the pheromone plume is very temporally complex, with EAGs exhibiting little activity until a pheromone odor was placed 1 m from the position of the EAG setup (A, B, C). Spectral analysis of the time series of EAG fluctuations revealed the strongest spectral intensities at frequencies of 0.8-1.8 Hz which corresponded to the EAG deflection rate of ca. 1.3 deflections/s (C). The EAG deflection rate was significantly lower at a distance close (25 cm) to the pheromone source (Kruskal-Wallis test with post-hoc Tukey multiple comparisons: χ_3,31_ = 21.85, *P* < 0.001) in comparison to the deflection rates 100 and 300 cm from the pheromone source which were not significant different from one another (Tukey test: *P* > 0.05). A similar trend was found for the smoke (TiCl_4_) tracer, where time series analysis of the smoke 1 m from the source showed a temporally complex signal (D) that had peak intensities in the 0.9-2.4 Hz frequencies (E) and mean filament frequencies of 1.9-2.4/s with increasing distance from the source (F). Finally, 3D sonic anemometry of the turbulent fluctuations in the wind tunnel revealed most of the turbulent energy being in the principal (*u*) axis (G, H, I) with increasing turbulent fluctuations with distance from the odor source.

**Supplemental Figure 5**

**
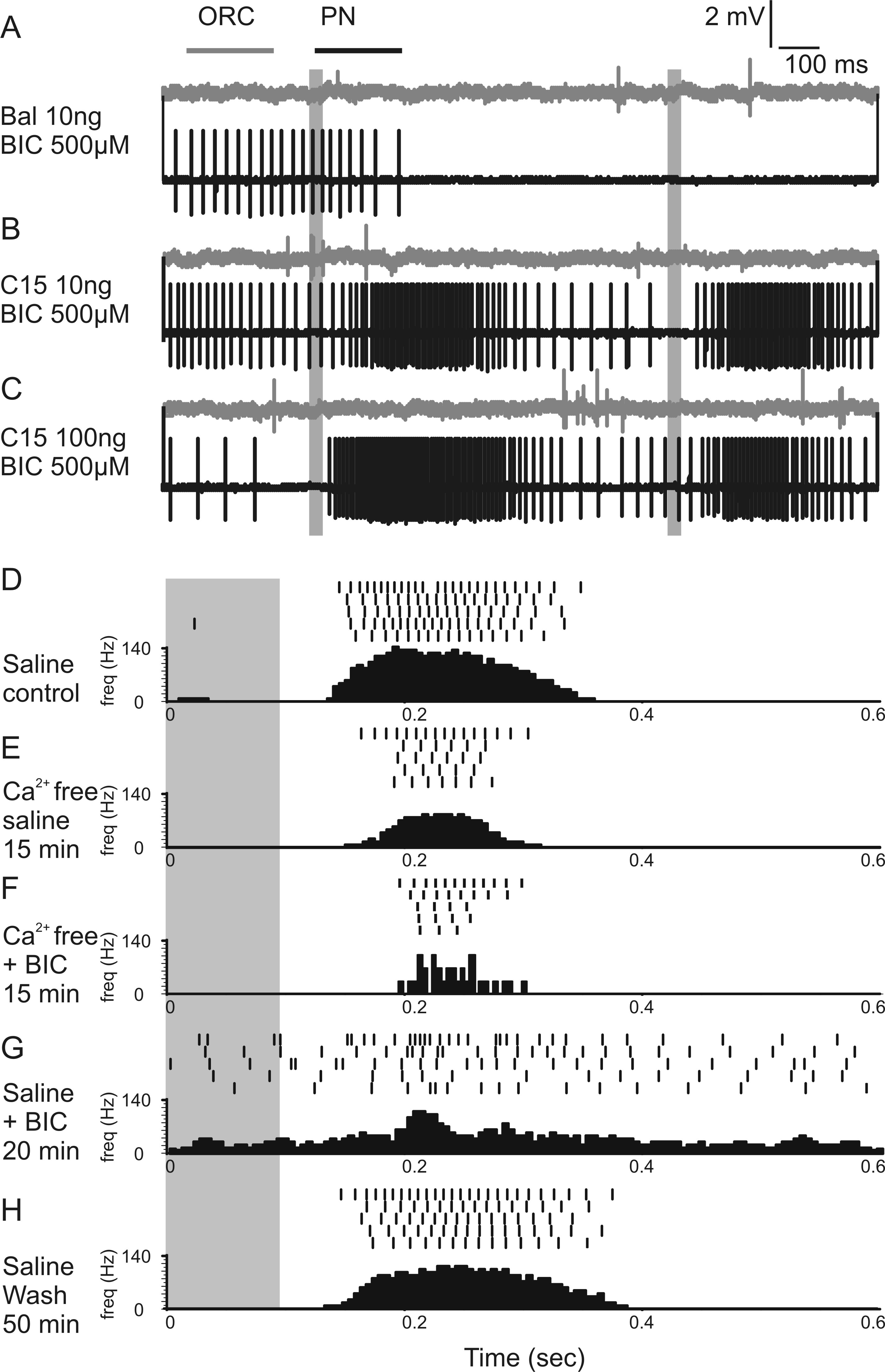
**

**Supplemental figure 5**. **Bicuculline (BIC) effects on MGC-PNs are not originated from the olfactory receptor neurons (ORC) and are calcium dependent**

(A-C) Simultaneous ORC and PN recordings showing that bicuculline application did not alter ORC’s spiking pattern. Different spike amplitudes are indicative of multiple ORCs housed in the same sensillum (grey traces; Trichoid Sensillum Type I [24]). The recorded C-PN displayed the typical bicuculline-induced tonic spontaneous firing (A) and prolonged excitatory response (lacking the I_2_ period) evoked by C-PN’s specific ligand C15 at two different concentrations (B, C) (black traces). The grey vertical bars indicate the two consecutive odor pulses. Notice that Bal, as the specific ligand for T-PNs, suppressed the C-PN’s spiking activity (A). Due to a higher threshold (>=100 times) ORCs generally do not respond at the concentration that triggers PN’s response [46].

(D-H) Peristimulus histograms demonstrating Ca^2+^ dependency of BMI effects on a different C-PN. The raster plots above the histograms show the aligned spiking response evoked by 5 consecutive odor pulses. The vertical shaded bar indicates odor stimulation. This PN displayed typical bursting response followed by silent I_2_ period evoked by its specific ligand C15 (100 ng) under saline control (D). This response pattern was maintained after replacing the normal saline with Ca^2+^ free saline for 15 min. However, the response duration and firing rate were reduced (E). Replacing the Ca^2+^ free saline with 500 μM BMI solution (diluted in Ca^2+^ free saline) for 15 min did not induce the typical bicuculline effects, i.e. tonic spontaneous firing and prolonged excitation, which would have occurred within this time frame if the drug was diluted in normal saline. Instead, the response magnitude was further reduced (F). After this trial, superfusing the preparation again with 500 μM bicuculline (but diluted in normal saline) for 20 min resulted in tonic spontaneous firing and prolonged excitatory response (G). Finally, washing with normal saline reversed the response pattern to control condition (H).

**Supplemental Figure 6**

**
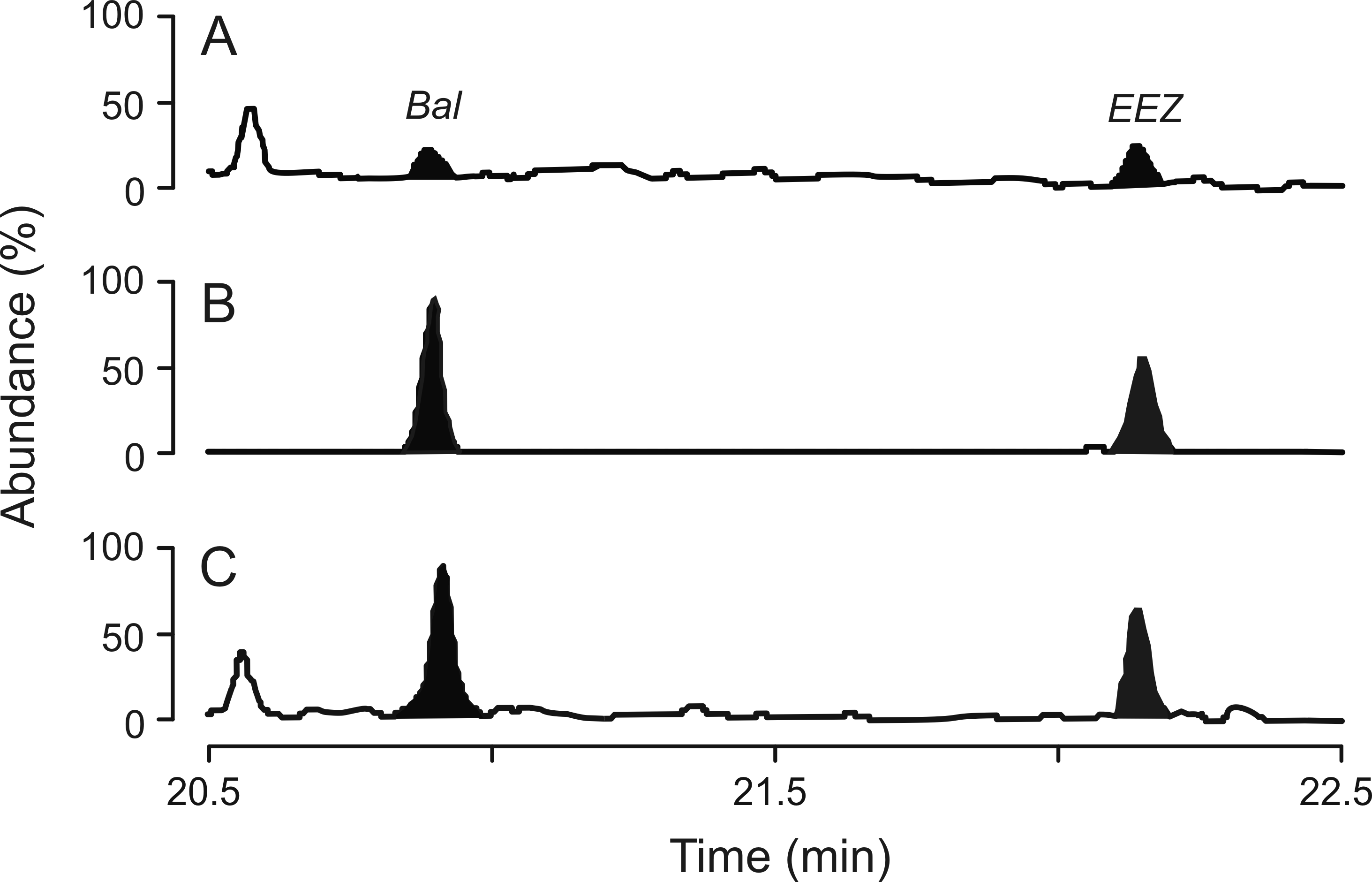
**

**Supplemental figure 6 Analytical GCMS comparison of the natural pheromone and a synthetic standard of the two pheromone components, (E,Z)-10,12-hexadecadienal (Bal) and (E,E,Z)-10,12,14-hexadecatrienal (EEZ).** (A) A sample of the female *M. sexta* headspace extract corresponding to 18 and 11 ng of Bal and EEZ, respectively, showing the diagnostic retention time of the sample. (B) 50 ng synthetic standards of Bal and EEZ showing the purity of the sample. (C) Co-injection of previous sample with the 50 ng synthetic Bal and EEZ standards, showing co-elution occurring as only 2 single peaks.
